# Fractal Analysis of BOLD Time Series in a Network Associated With Waiting Impulsivity

**DOI:** 10.3389/fphys.2018.01378

**Published:** 2018-10-04

**Authors:** Atae Akhrif, Marcel Romanos, Katharina Domschke, Angelika Schmitt-Boehrer, Susanne Neufang

**Affiliations:** ^1^Center of Mental Health, Department of Child and Adolescent Psychiatry, University of Wuerzburg, Wuerzburg, Germany; ^2^Department of Psychiatry and Psychotherapy, Faculty of Medicine, Medical Centre - University of Freiburg, Freiburg, Germany; ^3^Center of Mental Health, Department of Psychiatry, Psychosomatics and Psychotherapy, University of Wuerzburg, Wuerzburg, Germany

**Keywords:** fMRI, Hurst Exponent, frontal cortex, nucleus accumbens, biomarker, impulse control disorders

## Abstract

Fractal phenomena can be found in numerous scientific areas including neuroscience. Fractals are structures, in which the whole has the same shape as its parts. A specific structure known as *pink noise* (also called fractal or 1/f noise) is one key fractal manifestation, exhibits both stability and adaptability, and can be addressed via the Hurst exponent (*H*). FMRI studies using *H* on regional fMRI time courses used fractality as an important characteristic to unravel neural networks from artificial noise. In this fMRI-study, we examined 103 healthy male students at rest and while performing the 5-choice serial reaction time task. We addressed fractality in a network associated with waiting impulsivity using the adaptive fractal analysis (AFA) approach to determine *H*. We revealed the fractal nature of the impulsivity network. Furthermore, fractality was influenced by individual impulsivity in terms of decreasing fractality with higher impulsivity in regions of top-down control (left middle frontal gyrus) as well as reward processing (nucleus accumbens and anterior cingulate cortex). We conclude that fractality as determined via *H* is a promising marker to quantify deviations in network functions at an early stage and, thus, to be able to inform preventive interventions before the manifestation of a disorder.

## Introduction

Fractal structures possess the property that the whole structure consists of parts, which have the same pattern composition but at different scales and/or in different sizes [e.g., broccoli, the Koch snowflake (Koch, 1904, 1906; Mandelbrot, 1967, 1983)]. Fractals can be found not only in static objects but also dynamic processes. This property of *self-similarity*, or in the temporal domain *scale invariance* (Suckling et al., 2008; Ivanov et al., 2009; Nagy et al., 2017) means that both, rapidly occurring changes and slowly proceeding dynamics follow the same structure, or better, that measures of the patterns are independent of the sampling rate, used during data acquisition (Riley et al., 2012).

For time series, this property is mathematically expressed as follows:

(1) $$\begin{array}{l} S\left(f\right)=\frac{C_{f}}{|f{|}^{\beta }} \end{array}$$

(2) $$\begin{array}{l} \beta =2H-1 \end{array}$$

**Figure 1:**
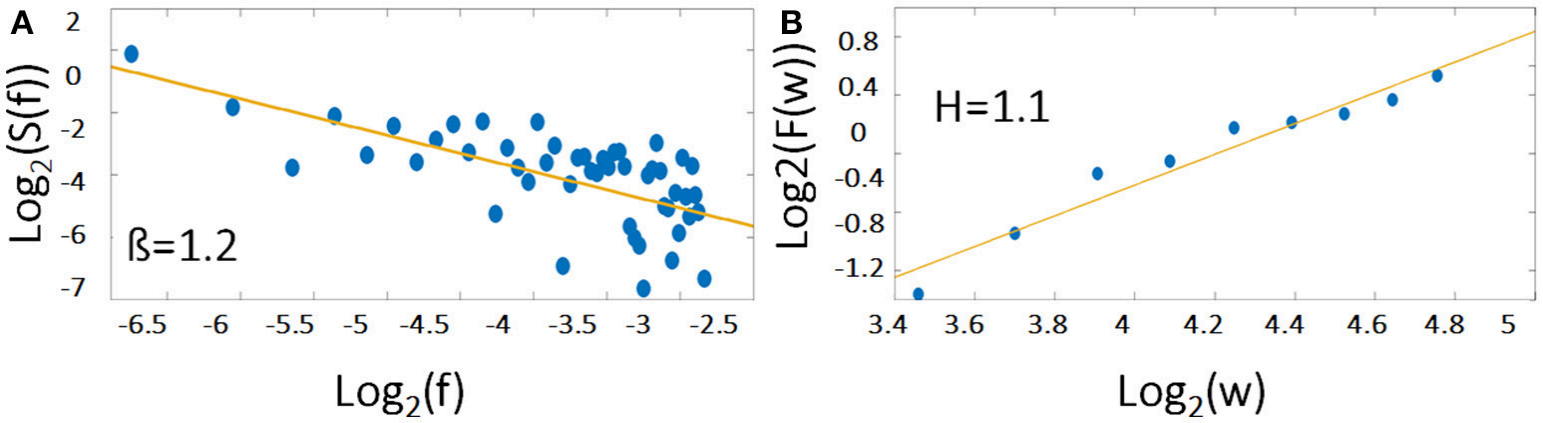
The 1/f noise pattern in a power spectrum (Equation 1) of an exemplary time course is shown on logarithmic scales while the scale invariance relation (Equation 3) is indicated by the slope *H*. Please note, that the relationship between β and *H* is according to Equation (2) β = 2*H* − 1. For demonstration, the example of a representative individual time course of the right MFG during task has been used.

*S*(*f*) represents the power spectrum density of the analyzed fluctuations, *f* the frequency, *C*_*f*_ a constant and 0 < β < 2. Furthermore, β is related to the Hurst Exponent (*H*) according to Equation 2 (see Figure 1). For more details on how to compute *H* refer to method section fractal analysis (AFA) as suggested by Riley et al. (2012).

Fractal patterns have been examined in many research fields including physiology and neuroscience. A specific phenomenon called *pink noise* (also called fractal or 1/f noise, with β = 1) is one of the key fractal manifestations. Pink noise is a stochastic process, used for the modeling of dynamic systems. Its power spectral density is inversely proportional to the sample frequency (Keshner, 1982; Eke et al., 2000, 2002). Because pink or 1/f noise lies between white noise ($\frac{1}{f^{0}}$, or random noise), and red/Brownian noise ($\frac{1}{f^{2}}$, power density decreases with increasing frequency), it has been proven to bring stability and adaptability into dynamic processes, thus, crucial properties of well-functioning complex systems (Bak et al., 1987). Pink noise has been documented in behavioral as well as physiological processes, for example in heartbeat dynamics (Ivanov et al., 1999, 2001), neural network organization (Lipsitz and Goldberger, 1992; Lipsitz, 2002) and cognitive processes (Ihlen and Vereijken, 2010; Wijnants et al., 2012). The manifold appearance of pink noise has led to the speculation, that “there exists some profound law of nature that applies to all non-equilibrium systems and results in such noise” (Sejdić and Lipsitz, 2013). Intuitively, one might assume that pink noise has a detrimental effect to a system's performance and accuracy. However, as pink noise arises from the interaction of multiple systems and operates over different scales, it has been shown to contribute to system resiliency and structural integrity if individual components were lost or interrupted for example by age or disease (Lipsitz and Goldberger, 1992; Lipsitz, 2002). Thus, a fractal network structure, thus, qualifies a system to cope with stress or disturbances by adjusting specific components and fine tuning its responses (for a review see Sejdić and Lipsitz, 2013).

Pink noise can be found in the fMRI signal (i.e., the Blood-Oxygen-Level-Dependent, BOLD response) (Bullmore et al., 2009; He, 2011; Herman et al., 2011; Ciuciu et al., 2012, 2014; Eke et al., 2012; Churchill et al., 2016; Nagy et al., 2017). *H* valued close to 1 in the fMRI signal has been associated with a higher predictability of time series (Gentili et al., 2017), greater low-frequency power and, therefore, higher persistence over time (Ball et al., 2011), as well as highly complex and well attuned dynamics in the underlying network (Lipsitz and Goldberger, 1992; Goldberger et al., 2002). Likewise, it has been shown that deviation from pink noise in relevant parameters, independent of whether the changes occurred in the direction of white or red noise, was associated with neurological as well as psychiatric disorders (resting-state fMRI in Alzheimer's disease: Maxim et al., 2005; e.g., reaction time sequences in attention deficit/hyperactivity disorder: Gilden and Hancock, 2007).

In addition, fractality seem to be more pronounced in low compared to high frequencies. For example, Fox et al. (2007) reported 1/f noise in the fMRI signal (Fox et al., 2007), emphasizing that “spontaneous BOLD follow a 1/f distribution, meaning that there is an increasing power in the low frequencies.” (Fox and Raichle, 2007). In addition, Gentili et al. (2017) found brain regions where H as well as metrics of low-frequency oscillations (i.e., amplitude of low-frequency fluctuations, ALFF, fractional amplitude of low-frequency fluctuations, fALFF) had similar effects hinting toward strong relation between both measures (Gentili et al., 2017). In task-fMRI data, fractal noise of inactive voxels differed from those of active ones (Thurner et al., 2003). Recent resting-state fMRI-studies showed, that *H* correlated with personality traits such as anxiety (Gentili et al., 2015) and extraversion (Gentili et al., 2017) in regions of the default mode network, with response time in the inferior frontal gyrus (Wink et al., 2008) hinting toward an influence of personality traits and task performance on the persistence of network dynamics (Wink et al., 2008). Likewise, findings from task-fMRI studies reported that *H* decreased with task processing (Ciuciu et al., 2012) and cognitive effort (Barnes et al., 2009; Churchill et al., 2016) concluding that “task-related modulation of multifractality appears only significant in functional networks and thus can be considered as the key property disentangling functional networks from artifacts” (Ciuciu et al., 2012).

Impulsivity is a personality trait, which spans from normal manifestations, e.g., in life time situations where decision making under time pressure is required (Burnett Heyes et al., 2012), to pathological presentations, mirroring the psychiatric symptoms of “loss of control” and “impulse control disorder” associated for example with ADHD (e.g., Sebastian et al., 2013; for a recent review see Hinshaw, 2017). Waiting impulsivity (WI) is one form of impulsivity and is operationally defined as the tendency to premature responding, i.e., to respond before target onset. Thus, it involves the aspects of response inhibition and top-down control, mediated by motivational aspects and reward processing (Robinson et al., 2009; Voon et al., 2014). Its associated functional network consists of the dlPFC and the vmPFC representing impulse control (Mechelmans et al., 2017), the reward-perception-related NAcc, the ACC for the cognitive evaluation of the reward and hippocampus (HC) and amygdala (AMY) responsible for reward-based learning (Dalley et al., 2011). Impulsivity has been documented to affect on the behavioral performance of attentional functions, working memory, motor speed, and language processing (Hinshaw et al., 2002; Huang-Pollock et al., 2006; Solanto et al., 2007). In brain activation, high impulsive healthy subjects showed reduced activation in right dorsolateral prefrontal cortex (dlPFC) while performing a descision making task (Deserno et al., 2015), bilaterally in the ventral prefrontal cortex (vmPFC) during motor inhibition (Goya-Maldonado et al., 2010) as well as in the dlPFC and the hippocampus in aggressive impulsive subjects (Sala et al., 2011). At rest, impulsivity affected functional connectivity from resting-state fMRI in terms of less elaborated neural network architecture, e.g., lateral and medial prefrontal regions were isolated from reward associated regions such as the nucleus accumbens (NAcc) (Davis et al., 2013), connectivity between the NAcc and the anterior cingulate cortex (ACC) as well as the ACC and the amygdala (Li et al., 2013). A first study addressing the influence of impulsivity on *H* revealed that impulsivity correlated negatively with *H* in the orbito-frontal cortex (i.e., the vmPFC) and NAcc (Hahn et al., 2012) the way that the higher impulsive the subjects the smaller the *H*.

In this study, we examined the fractal nature of a brain network associated with WI using the AFA approach. *H* was determined for all network regions at rest and while performing a WI task. To define, whether a subject is high (highImp) or low impulsive (lowImp), the number of premature responses has been used (Feja et al., 2014; e.g., Donnelly et al., 2014). A permutation test was performed to insure the validity of using the number of premature responses as grouping criteria (see Supplement permutation.xlsx—Supplementary Datasheet 1). Based on the introduced findings we were intrigued to address the existence of pink noise in our network, thus, we expected to find

- a fractal nature of the impulsivity network and that fractality consists of pink noise, i.e., *H* values of all network regions were close to 1.
- smaller *H* at task compared to rest (Barnes et al., 2009; Ciuciu et al., 2012; Churchill et al., 2016).
- significant influence of impulsivity on *H* predominantly in the PFC and the NAcc (Wink et al., 2008; Hahn et al., 2012). In line with the previous studies (Gilden and Hancock, 2007; Hausdorff, 2007) we expected to find deviation from 1/f noise pronounced in highImp compared to lowImp subjects.

## Materials and methods

### Subjects

In this study, data of the same sample of 103 students was used as described by Neufang et al. (2016). Students were between 19 and 28 years old (24.0 ± 2.6 years), and were recruited at the University of Wuerzburg, Germany. From all subjects, measures for impulsivity were collected, using the Wender-Reimherr-Interview and Attention-Deficit/Hyperactivity Disorder checklist (subscales “impulsivity” and “hyperactivity and impulse control”) (Rösler et al., 2008). The examination was conducted in accordance with the Declaration of Helsinki in its latest version from 2008 and was approved by the ethics committee of the Faculty of Medicine, University of Wuerzburg, and written informed consent was obtained from all subjects.

### Experimental paradigm

**Figure 2:**
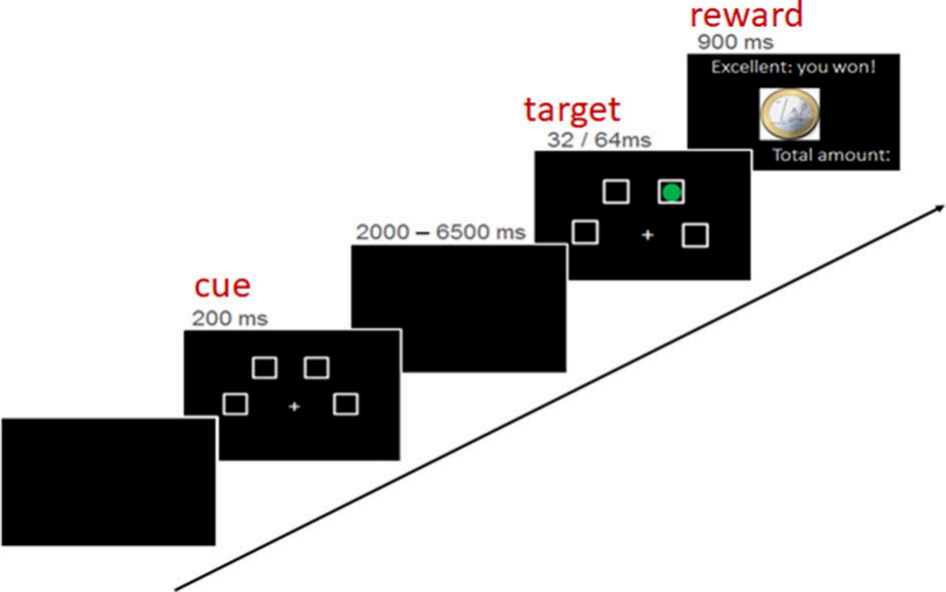
Structure of an experimental trial.

The used paradigm was an fMRI-adaptation of the four-choice serial reaction time task by Voon et al. (2014) and has been described in detail by Neufang et al. (2016), Voon et al. (2014), and Neufang et al. (2016). The task entailed the detection of a brief visual target (a green dot) after a certain waiting period. Depending on the subject's task performance, a reward of 1 Euro or 1 Cent was given, or better the punishment of 1 Euro was subtracted. In detail, a trial implied three phases/experimental conditions: the “cue” presentation, indicating the start of the waiting period; the “target” onset, in terms of a green circle in one of the choices. Subjects were instructed to indicate the correct choice by pressing the corresponding button as fast and as correct as possible; the reward feedback, showing the amount of recently earned/lost money in combination with the overall amount of earned money (see Figure 2).

The task consisted of one baseline run outside the scanner and five experimental runs within the scanner. During the five runs in the scanner, WI was experimentally manipulated by (i) implementing a monetary reward (blocs 1, 3, 4, 5), (ii) varying the duration of the target presentation (blocs 3, 4, 5), (c) extending the waiting period (cue-target interval) (blocs 4 and 5) and (d) presenting additional distractor taget before the actual target presentation, i.e., circles in different colors (bloc 5). Task scanning was of a total duration of 14 min.

Before task-fMRI, all subjects were scanned at rest for 12 min. During the rs-fMRI, subjects kept their eyes open and were instructed not to think of something specific. Eyes were controlled by the examiner via an eye tracking camera outside the MR scanner.

### fMRI—data acquisition

The fMRI scanner was a 3 Tesla TIM Trio Scanner (Siemens, Erlangen, Germany). Functional MRI included a T2^*^-weighted gradient echo-planar imaging sequence with the following sequence parameters: repetition time (TR) = 2000 ms, echo time (TE) = 30 ms, 36 slices, 3 mm thickness, field of view (FoV) = 192 mm, flip angle = 90°, number of volumes in task-fMRI = 425, number of volumes in rs-fMRI = 350).

### fMRI—data processing

Data preprocessing was performed using the Statistical Parametric Mapping Software Package (SPM12). Preprocessing followed the standard routine including temporal and spatial alignment, i.e., slice time correction and realignment and unwarp, spatial normalization [standard space: Montreal Neurological Institute (MNI) space] including a resampling of the data to an isotropic voxel size of 2 × 2 × 2 mm^3^, spatial smoothing with a Gaussian kernel of 8 mm full width at half maximum (FWHM), and linear trend removal [using the matlab routine detrend (y)] (see Supplementary Presentation 1) (Bai et al., 2008; Zhang et al., 2008; Fox et al., 2009; Qiu et al., 2011). Pre-processing did not include high-pass filtering or global mean correction.

### fMRI time course extraction

Regions of Interest (ROI) were defined based on the significantly activated brain regions while performing the waiting impulsivity task. In detail, for the identification of global activation maxima, the contrasts *target* > *baseline* and *reward* > *baseline* were defined on a single subject level and analyzed on group level using a one sample *t*-test. The local maxima of each significantly activated regions were identified and coordinates were then used as the centere of a 10 mm spheric ROI using MarsBar [24]. ROIs were built and used for the extraction of the time course for each subject. Time course extraction was performed using the routine as suggested by Brett et al. (2002) (see MarsBar manual, http://marsbar.sourceforge.net/ marsbar.pdf) from preprocessed fMRI data (i.e., smoothed files resulting from the pre-processing procedure) (Brett et al., 2002).

### AFA

**Figure 3:**
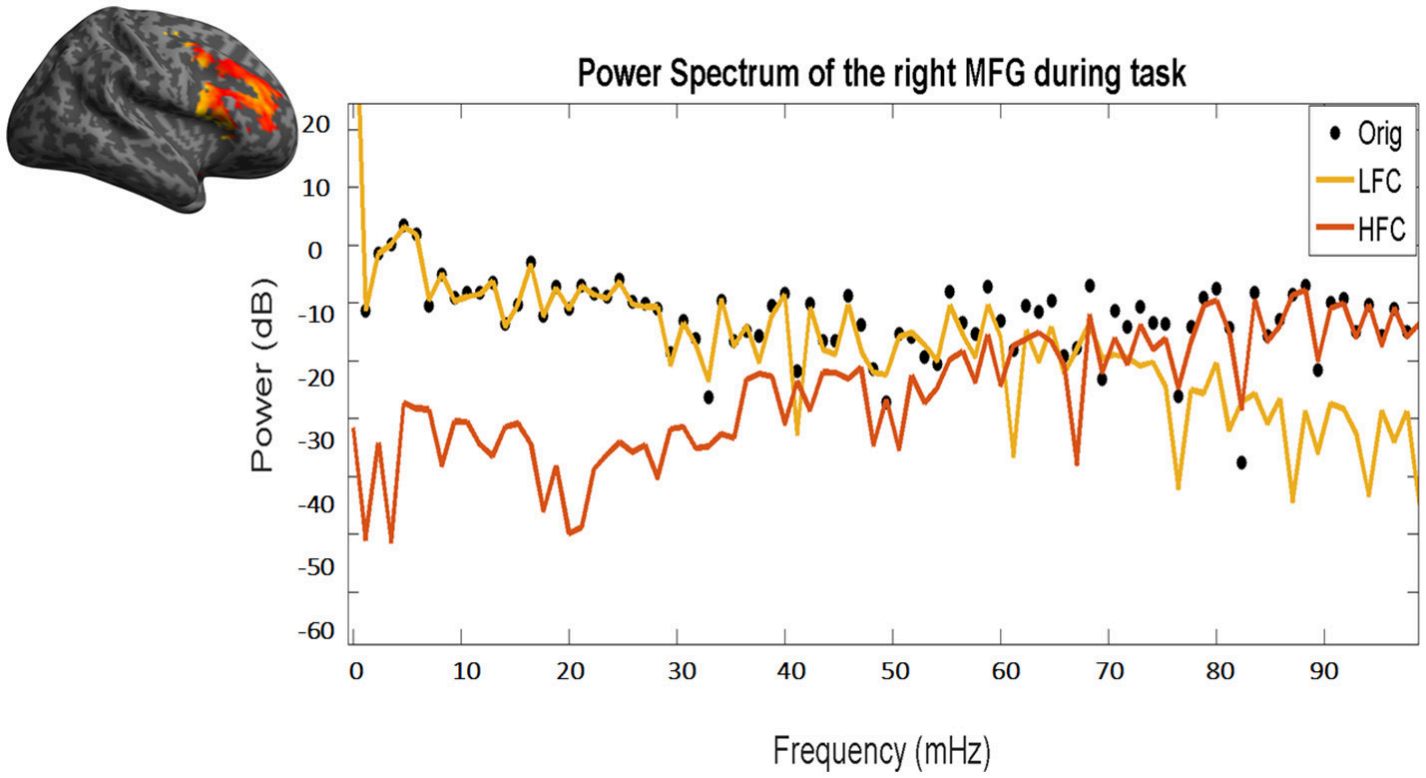
Power frequency spectrum of the original time course (black dots), as well as of the resulting low (yellow line) and high frequency (red line) components. Please note the overlap between the black dots (original time course) and the yellow line (LFC) in low frequencies (left part of the x-axis) and between the black dots and the red line (HFC) in high frequencies (right part of the x-axis). The same time course of the right MFG during task of a representative subject has been used.

Fractal analysis of time series is based on quantifying the degree of fluctuation around the overall trend of the data over time, to measure the scale invariance quantified by the value of *H* (Equation 3, see below). In this paper, we split the fMRI signal in its two components: the low and the high frequency components (LFC, HFC) (see Figure 3).

**Figure 4:**
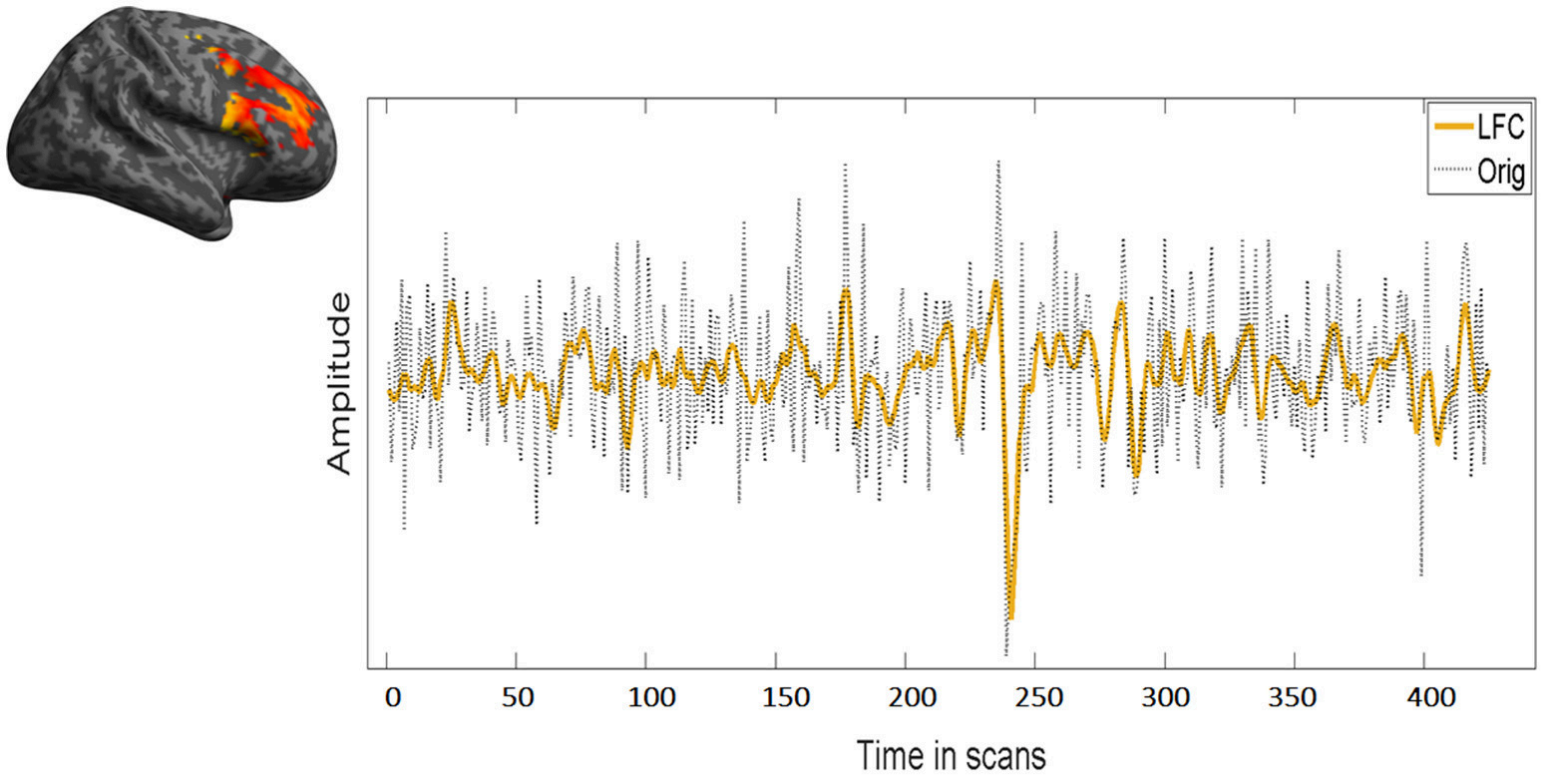
Overlap between low frequency course (LFC) and the original time course. LFC fits well the data without overfitting leaving out unnecessary information for the fractal analysis. In this figure, the same time course of the right MFG during task of a representative subject has been used.

**Figure 5:**
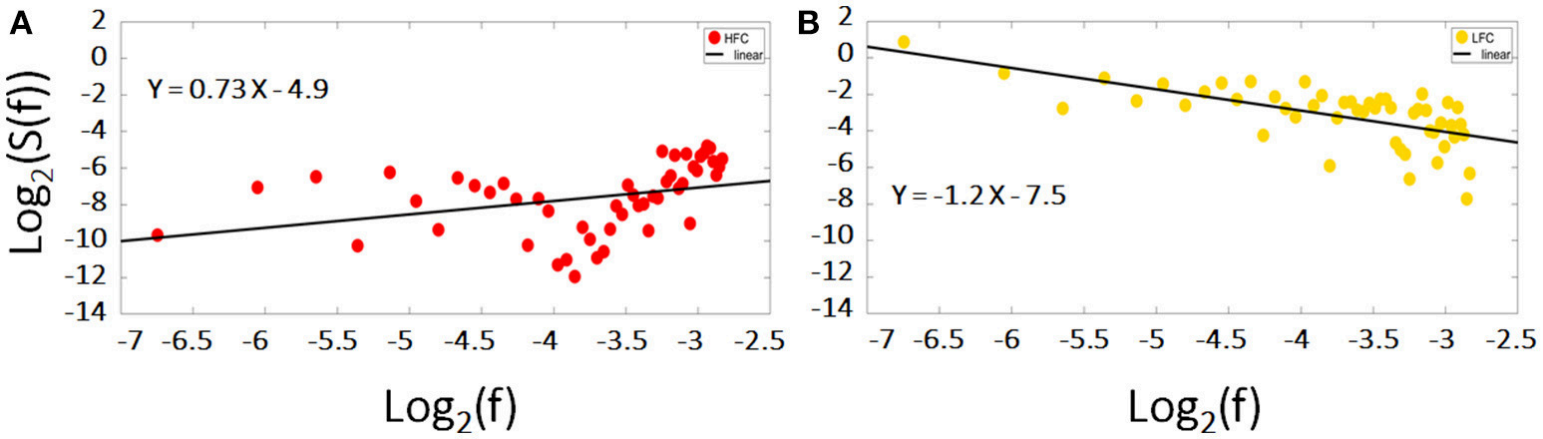
Log-log diffusion plots of the power spectrum density (PSD) of the low and high frequency components (LFC, HFC): whereas the HFC (red dots) fails to fulfill the criteria of a fractal structure expressed by Equation 1, the PSD of the LFC (yellow dots) is clearly inversely proportional to frequency hinting toward a fractal nature. The data presented is the same time course of the right MFG during task of a representative subject.

LFC is the second order polynomial that is a smooth and global fit of the original time course (see Figure 4). HFC represents the residuals after subtracting the fitting curve from the original time course. For time series to be fractal, their power spectrum density (PSD) must be inversely proportional to frequency (see also Figure 5, legend).

After analyzing these two main components, we found, that the residuals were more likely to obscure the results with respect to the scale invariance analysis. HFC, in addition, could not be classified as fractal (see Figure 5A). The low frequency component of the signal on the other hand, held all the information concerning the fractal nature of the original signal. In line with earlier studies and to avoid inaccuracies and the reduction of *H*, we focused in our analyses on those parts of the signal/those regions, which showed power law scaling and fractal scaling was present (e.g., Cannon et al., 1997; Herman et al., 2011; Riley et al., 2012): only the LCF was taken into consideration for further analysis via AFA to compute *H*.

AFA is one of the existing mathematical methods that computes *H*, a factor that reflects in a scale law manner the relationship, that is intrinsic to fractal processes, between the variance of fluctuation computed around, in our case, a second order polynomial trend *v*(*i*) fitted to time series within each segment *w*, and its size:

(3) $$\begin{array}{l} F\left(w\right)={\left[\frac{1}{N}\overset{N}{\underset{i=1}{\sum }}{\left(u\left(i\right)-v\left(i\right)\right)}^{2}\right]}^{\frac{1}{2}}\sim w^{H}, \end{array}$$

*N*: length of the time series

$$\begin{array}{l} w=2n+1,\;n=5,6\ldots ,13 \end{array}$$

*H* is determined as the slope of the log-log diffusion plot log_2_ (*F*(*w*)) as a function of log_2_ (*w*) (see Figure 1B).

### Statistical analysis

The factor *impulsive phenotype* was defined as high impulsive (highImp) versus low impulsive subjects (lowImp), based on the subjects' number of premature responses. If the number was ≥ 3 they were classified as highImp and if the number was <3 as lowImp. Threshold definition was adapted from Feja et al. (2014) in terms of the median value of premature responses across all subjects [range: 0–6 number of premature responses (adapted from Feja et al., 2014)]. The sample consisted of 66 lowImp subjects and 38 highImp. In addition, a permutation test was performed to insure the validity of using the number of premature responses as grouping criteria (see Supplement permutation.xlsx—Supplementary Datasheet 1).

On behavioral level, 1 × 2 ANOVA models were defined using the between-subject factor *impulsivity phenotype* (highImp vs. lowImp) as independent factor and the dependent variables the behavioral parameters *no. of premature responses, accuracy, reward (amount of total win)* and *reaction times*.

The question of the existence of pink noise was verified using a one sample Wilcoxon test with *H* of all network regions as test variables and 1 as hypothetical median.

To address the two aspects (a) changes in fractality at rest and while task processing and (b) the influence of impulsivity on fractality of the impulsivity network, the following statistical analyses were performed:

- To compare fractality at rest and while task processing, non-parametric tests of related samples were defined using the within-subject factor *condition* (task vs. rest), and *H* as dependent variable. To reveal the impact of the impulsive phenotype on differences between rest and task, the same analyses were performed phenotype-specifically.
- The influence of impulsivity on network fractality was performed using both, the factorial and the dimensional approach. Impulsive phenotype differences of *H* were addressed using non-parametric Mann-Whitney-U-Test for 2 independent samples using the between-subject factor *impulsive phenotype* and dependent variables were *H* scores. In addition, correlations between WI (i.e., the number of premature responses, task accuracy, reward and reaction times) and *H* scores were performed.

For all statistical analyses a significance threshold of *p* < 0.05, corrected for multiple comparisons using the False-Discovery Rate (Benjamini and Hochberg, 1995), was applied. The number of test as well as q^*^-scores representing the FDR-corrected significance levels were provided for each analysis in the results section as well as in Tables 1, 2.

**Table 1:** Comparison of H between task and rest across all subjects.

	**Across all subjects**			**lowImp**			**highImp**		
	**Task [M(SD)]**	**Rest [M(SD)]**	**Z**	**Task [M(SD)]**	**Rest [M(SD)]**	**Z**	**Task [M(SD)]**	**Rest [M(SD)]**	**Z**
rHC	0.88 (0.11)	0.93 (0.13)	3.7^**^	0.88 (0.11)	0.94 (0.13)	3.4^**^	0.88 (0.11)	0.92 (0.12)	n.s.
lHC	0.90 (0.10)	0.96 (0.12)	3.8^**^	0.91 (0.10)	0.96 (0.13)	2.6^**^	0.88 (0.10)	0.95 (0.10)	2.8^**^
lMFG	0.93 (0.11)	1.01 (0.12)	5.4^**^	0.94 (0.11)	1.02 (0.11)	4.1^**^	0.90 (0.12)	1.00 (0.12)	3.5^**^
rMFG	0.92 (0.13)	1.00 (0.12)	4.4^**^	0.93 (0.14)	1.00 (0.13)	2.7^**^	0.90 (0.12)	1.00 (0.12)	3.6^**^
ACC	0.93 (0.12)	1.01 (0.12)	4.8^**^	0.96 (0.13)	1.02 (0.13)	2.8^**^	0.89 (0.09)	0.99 (0.10)	4.1^**^
rNAcc	.91 (0.13)	0.97 (0.13)	3.6^**^	0.93 (0.13)	0.98 (0.12)	2.3^**^	0.87 (0.13)	1.00 (0.12)	2.9^**^
lAMY	0.88 (0.11)	1.02 (0.12)	3.0^**^	0.89 (0.12)	0.92 (0.12)	n.s.	0.86 (0.11)	0.95 (0.14)	2.7^**^
vmPFC	0.98 (0.11)	1.07 (0.12)	5.4^**^	0.98 (0.11)	1.07 (0.12)	4.2^***^	0.97 (0.11)	0.91 (0.12)	3.3^**^

rHC, right hippocampus; lHC, left hippocampus; lMFG, left middle frontal gyrus; rMFG, right middle frontal gyrus; ACC, anterior cingulate cortex; Nacc, nucleus accumbens; lAMY, left amygdala; vmPFC, ventromedial prefrontal gyrus; lowImp, low impulsive subjects; highImp, high impulsive subjects; FDR-corrected was applied for 8 comparisons; corrected significance level were q^*^(all subjects) = 0.05; q^*^(highImp subjects) = 0.04; q^*^(highImp subjects) = 0.04;

** p < q^*^; n.s., not significant.

**Table 2:** Comparison of H between high and low impulsive subjects.

	**lowImp [M(SD)]**	**highImp [M(SD)]**	**Z**
**TASK**			
rHC	0.87 (0.11)	0.88 (0.11)	0.2
lHC	0.91 (0.10)	0.88 (0.10)	1.9
lMFG	0.94 (0.11)	0.90 (0.11)	1.7
rMFG	0.93(14)	0.90 (0.11)	1.1
ACC	0.96 (0.13)	0.89 (0.09)	3.0^**^
rNAcc	0.93 (0.13)	0.87 (0.13)	2.4^**^
lAMY	0.89 (0.11)	0.86 (0.11)	1.4
vmPFC	0.98 (0.11)	0.97 (0.11)	0.6
**REST**			
All regions	n.s.		

rHC, right hippocampus; lHC, left hippocampus; lMFG, left middle frontal gyrus; rMFG, right middle frontal gyrus; ACC, anterior cingulate cortex; Nacc, nucleus accumbens; lAMY, left amygdala; vmPFC, ventromedial prefrontal gyrus; _low_, low impulsive subjects; _high_, high impulsive subjects; FDR-corrected was applied for 16 comparisons; corrected significance level was q* = 0.007;

** p < q*; n.s., not significant.

## Results

*post-hoc* power analyses using G^*^Power (version 3.1.9.3, http://www.gpower.hhu.de/) revealed a power of 0.77 and a critical *Z* = 1.6.

Regarding behavioral differences only main effect of impulsive phenotype on the number of premature responses passed the threshold of significance (Ml_owImp_ = 0.6 ± 0.5, M_highImp_ = 2.5 ± 0.9, *F*_(102, 2)_ = 201.8, *p* = 0.000, corrected for 4 comparisons with q^*^ = 0.0125). All other effects on any other dependent variable were not significant (for further results please see Neufang et al., 2016).

**Figure 6:**
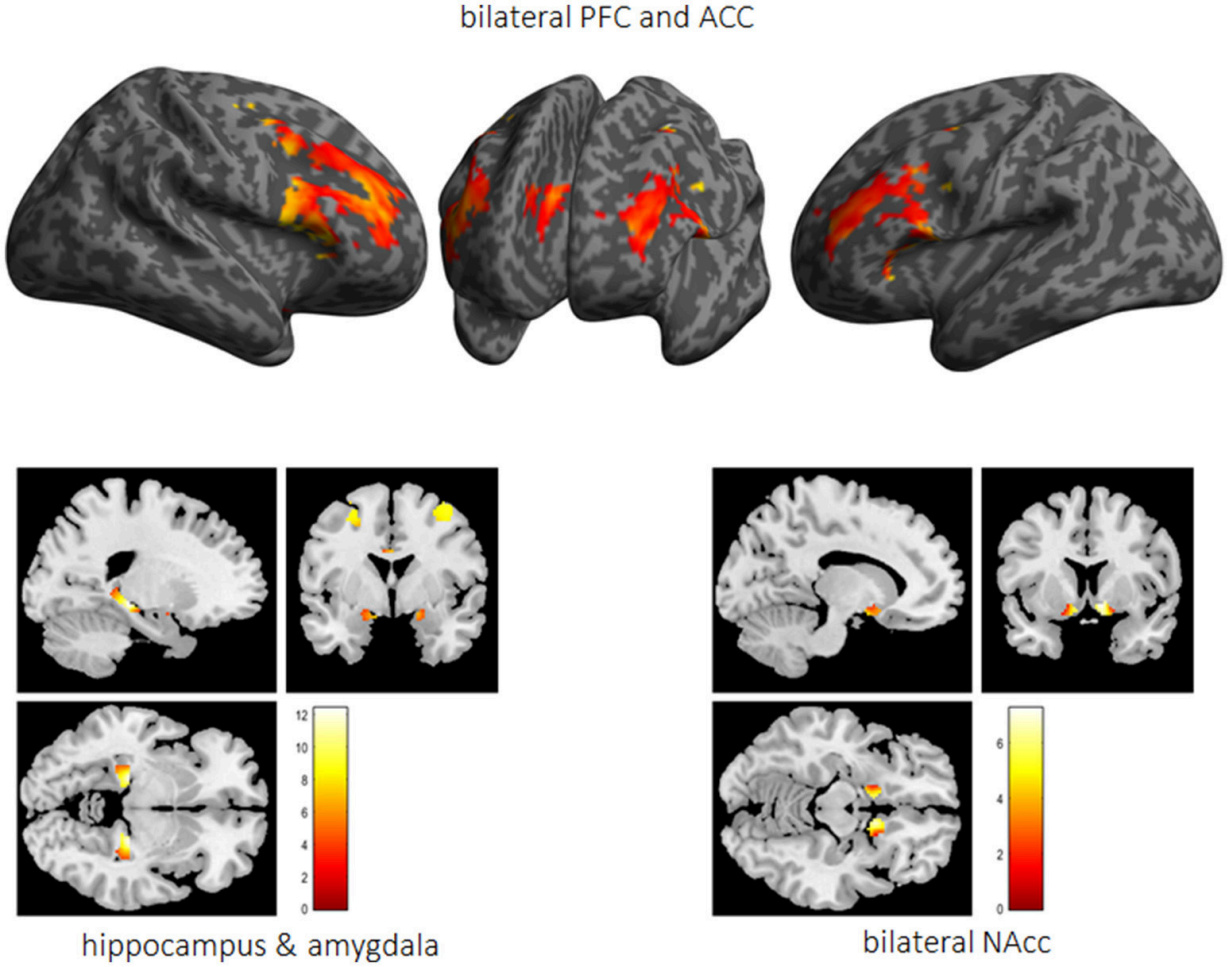
The impulsivity network is presented in terms of significantly activated brain regions across all subjects while performing the 5-choice serial reaction time task. PFC, prefrontal cortex; ACC, anterior cingulate cortex; Nacc, nucleus accumbens.

Performed fMRI analyses revealed that whereas activation bilaterally in the middle frontal gyrus (MFG) (right MFG: *x* = 40, *y* = 0.8, 34, *T* = 19.7; left MFG: *x* = −44, *y* = 6, 28, *T* = 21.7), the ACC (*x* = 6, *y* = 30, 28, *T* = 18.6) as well as the vmPFC (*x* = 0, *y* = 48, −12, *T* = 6.5) was associated with impulse control, bilaterally the HC (right HC: *x* = 24, *y* = −28, −6, *T* = 21.9; left HC: *x* = −22, *y* = −28, −6, *T* = 17.7), the right NAcc (*x* = 8, *y* = 12, −10, *T* = 14.6), and the left amygdala (*x* = −22, *y* = 0, −12, *T* = 6.0) were active while reward processing (see Figure 6).

Via AFA we found that at rest (a) across all subjects, *H* was similar to 1 in the following network regions (rHC: M = 0.93 ± 0.13, *p* = 0.000; lHC: M = 0.96 ± 0.12, *p* = 0.000; lMFG: M = 1.01 ± 0.12, *p* = 0.156; rMFG: M = 1.00 ± 0.12, *p* = 0.577; ACC: M = 1.01 ± 0.12, *p* = 0.414; rNAcc: M = 1.03 ± 0.10, *p* = 0.060; lAMY: M = 1.00 ± 0.11, *p* = 0.928; vmPFC: M = 1.01 ± 0.12, *p* = 0.087, corrected for 8 comparisons with q^*^ = .006) proving a stable fractal nature of this network for confidence intervals from bootstrap see Supplementary Table 1.

- Across all subjects (i.e., independent of the impulsive phenotype), *H* was significantly higher at rest compared to task in all regions. Group-specific analyses, however, revealed that in highImp subjects, fractality in the right HC did not differ at rest and during task processing (see Table 1). In lowImp subjects, *H* was significantly higher at rest compared to task in all regions.
- *H* during task-processing differed between impulsivity phenotypes in terms of reduced *H* in highImp subjects in the reward-associated NAcc and the impulse control-related ACC (see Table 2). Furthermore, *H* of the left HC varied trend-wisely between impulsivity groups across. At rest, there was no significant difference in any region. Correlations revealed a significant correlation between *H* of the left MFG and the number of premature responses (*r* = −0.242, *p* = 0.013, corrected for 16 comparisons with q^*^ = 0.012), whereas there was no significant relation between *H* and accuracy, reward and reaction times.

## Discussion

In this study, we addressed the fractal nature of a neural network associated with waiting impulsivity. We found that (a) pink noise in all network regions, proving the existence of a stable fractal nature within this network (Bak et al., 1987; Lipsitz and Goldberger, 1992; Lipsitz, 2002; Wijnants et al., 2012). Furthermore, (b) *H* was significantly higher at rest compared to task. This was the case in all regions and across all subjects. However, in high impulsive (highImp) subjects, *H* was comparable between both activation conditions in the right HC. (c) Finally, during task processing, fractality in impulse control related left MFG as well as reward-associated NAcc and ACC was influenced by impulsivity the way that in highImp subjects *H* was significantly smaller and, therefore, was a less adequate 1/f noise fit candidate compared to lowImp subjects.

### The fractal nature of the waiting impulsivity network

As introduced we learned that a brain network follows a stable fractal patterns when *H* is close to 1 (e.g., Stadnitski, 2012), is decreased while cognitive activation (Barnes et al., 2009; Ciuciu et al., 2012; Churchill et al., 2016), and is sensitive to normal and abnormal alteration such as disease or age (Lipsitz and Goldberger, 1992; Lipsitz, 2002; Sejdić and Lipsitz, 2013). In our data, we could find all these aspects and, hence, assumed a fractal nature in the impulsivity network: (a) at rest, *H* varied around 1 across all subjects; during task processing, *H* was significantly reduced in all regions and, finally, when comparing *H* between high and low impulsive subjects, *H* was reduced in highImp subjects in some of the network regions. The network examined here has been introduced in a comprehensive review article by Dalley et al. (2011) and is based on relevant findings from humans and animal studies on impulsivity and cognitive top-down control (Dalley et al., 2011). The notion that the suggested network regions were indeed involved in the processes of waiting impulsivity as measured via the 5-CSRTT has been shown in recent studies (NAcc and ACC: Morris et al., 2016; e.g., NAcc and vmPFC: Neufang et al., 2016; dlPFC and ACC: Mechelmans et al., 2017). The characterization of this network as a “healthy and complex system” (Bak et al., 1987), however, has been demonstrated in this study for the first time.

### Fractality during task processing and at rest

Higher fractality at rest compared to task processing/cognitive effort is in line with earlier findings (He, 2011; Ciuciu et al., 2012; Churchill et al., 2016). In the context of the common knowledge, that neural networks are predominantly active after an external stimulation, e.g., of our senses or while cognitive processing (Penn and Shatz, 1999; Kandel et al., 2000), this finding seems counter-intuitive. However, the recent years of research on the brain at rest have accentuated the prominence of *endogenously* engendered brain responses as an important defining factor in modeling the topology of large-scale neuronal networks (for reviews see Linkenkaer-Hansen, 2002; Calhoun and De Lacy, 2017; Gorges et al., 2017; Liégeois et al., 2017; Smitha et al., 2017). The terms used most frequently to describe resting-state neural activity, such as “endogenous,” “intrinsic” and “spontaneous,” indicate that network function is created within the brain itself, and can, thus, be understood as “self-organized” (Linkenkaer-Hansen, 2002). Self-organized criticality, in return, has been described by Bak et al. (1987) as the origin of fractal objects. They demonstrated, that “dynamic systems naturally evolve into self-organized critical structures of states” and suggested, that “this self-organized criticality is the common underlying mechanism“ behind those dynamic system (Bak et al., 1987). For an empirical example in the context of sleep dynamics, Lo et al. (2013) were able to identify two independent paths for the transition between sleep phases using power-law scaling on noctural EEG recordings (Lo et al., 2013). Thus, a task-induced stimulation operating as an involvement from the outside system may lead to a reduction of these dynamics, hence of fractality.

In highImp subjects, however, *H* did not decrease significantly during task in the right HC. A generally small fractality in the hippocampus has been reported by He (2011, 2014). They found varying *H* across different cortical regions with lowest *H* in the HC, interpreting these findings in terms of regional differences in neurovascular coupling mechanisms (He, 2011, 2014;). Impaired hippocampal *H* has also been reported between patients with Alzheimer's disease and control subjects (Maxim et al., 2005) with the persistence of *H* being assumed to reflect neurodegenerative processes. The insignificant change of *H* during task processing in high impulsive subjects in our study, thus, might reflect a weaker recruitment of the right HC while performing the task and a more superficial learning (e.g., El-Gaby et al., 2015). In return, this finding can also be interpreted the way, that the right HC is less adaptive in high impulsive subjects, thus constantly following its own dynamics, leading to an impaired motivation- or reward-based learning of the task (Chantiluke et al., 2012; Moreno-López et al., 2012).

### Fractality differs in function of impulsivity

Our analyses revealed, that impulsivity modulated fractality only during task processing and predominantly in the fronto-striatal loop namely the ACC and the NAcc, as well as the left MFG. Morris et al. (2016) showed that functional connectivity in the ACC and the NAcc via the subthalamic nucleus varied in function of the number of premature responses (Morris et al., 2016) emphasizing the crucial and interacting role of these two structures on the key parameter of waiting impulsivity. The left MFG as part of the dorsolateral PFC, in return, reflects the counterpart, i.e., top-down control which decreases with higher reward processing (Mechelmans et al., 2017).

In the NAcc as well as in the ACC, in high impulsive subjects, *H* was significantly reduced compared to low impulsive subjects. A negative association between impulsivity/reward sensitivity and the ventral striatum has been reported by Hahn et al. (2012) before in the way that the higher impulsive/reward sensitive the subjects were, the smaller the *H* (Hahn et al., 2012). Reduced *H* in the NAcc and ACC in high impulsive subjects of our study, thus, reflects an altered reward processing.

In addition, significant correlations between impulsivity and fractality in the frontal cortex have been shown for the orbito-frontal cortex (Hahn et al., 2012) as well as for the lateral PFC (Ball et al., 2011). Similar to these findings, we found a significant (negative) correlation with the number of premature responses and *H* in the left MFG across all subjects as well as in the vmPFC in the highImp group. The MFG is strongly involved in response inhibition and cognitive control (Chambers et al., 2009; Boehler et al., 2010; Braver, 2012; Bari and Robbins, 2013), thus, a more random top-down control in highImp subjects reflects impaired control and more impulsive task performance. Taken together, the combination of impaired top-down control and altered reward processing is common and has been described for numerous impulse control disorders such as attention-deficit/hyperactivity disorder (e.g., Scheres and Hamaker, 2010), eating disorders (for review Citrome, 2015), addiction (Weinstein, 2017), bipolar disorder, and depression (Carver et al., 2008).

In contrast to findings by Wink et al., *H* of network regions associated with waiting impulsivity did not correlate with reaction time (Wink et al., 2008). This, however, might be based on the different anatomical structures: Wink et al. reported a correlation of *H* and resting-state fMRI signals in the right inferior frontal cortex, which was not included in our network.

## Limitation and conclusion

In this study, we chose a monofractal approach. The main reasons were the following: (1) This is a pilot study for a clinical project; thus, data acquisition was strongly determined by the factors (a) field strength (3T) (b) total scanning time (12 min/365 volumes during resting-state; 14 min/420 volumes during tast), (c) sample rate (TR was 2000 ms) to ensure the feasibility for patients to perform the scanning procedure successfully. All factors, however, play a crucial role in the analysis of fractal patterns in fMRI (Eke et al., 2012). For example, the field strength highly influences measurement sensitivity (Eke et al., 2006) and multi-fractal analysis is “known to require a much higher signal definition for an optimal performance” than monofractal (Ciuciu et al., 2012; Eke et al., 2012). Likewise, multi-fractal analyses need longer time series/higher sampling rates than those found for monofractal series (Eke et al., 2002, 2012). (2) In a previous step multi-fractality was addressed (using q from −2 to 2) and revealed, that our signals are, with no loss of information, to be approximated as monofractal (see Figure S1). The monofractal approximation, however, has been proven to be a robust assumption and, thus, an adequate tool to address similar signals.

In summary, we revealed that activity in the neural network associated with waiting impulsivity is of fractal nature. The use of fractal parameters to examine neural networks regarding to health or disorder has been described in earlier studies (e.g., Lipsitz, 2002; Maxim et al., 2005; Hahn et al., 2012; Lei et al., 2013; Sokunbi et al., 2014; El-Gaby et al., 2015; Dona et al., 2017; Gorges et al., 2017), introducing these parameters as exceptionally sensitive toward alterations. In our study, however, we performed analysis in a very homogenous sample of young adult male students. The classification into high and low impulsive subjects, therefore, is relative and does not represent samples with manifest impulse control disorders. The transfer of the present data to a clinical context therefore predominantly relies on the findings of earlier studies (e.g., Lipsitz, 2002; Maxim et al., 2005; Hahn et al., 2012; Lei et al., 2013; Sokunbi et al., 2014; El-Gaby et al., 2015; Dona et al., 2017; Gorges et al., 2017) and would be of high interest for future studies.

In contrast to earlier studies, in which *H* was determined on the whole brain level and in a data-driven manner (e.g., Suckling et al., 2008; Wink et al., 2008; Barnes et al., 2009; Gentili et al., 2015, 2017; Churchill et al., 2016), we chose to focus on an earlier described network. This way, we were able to *a priori* match cognition and neural structures, however, taking the risk of losing information, for example regarding the compensatory recruitment of additional structures. When examining a clinical sample, thus, a combined approach would be indicated.

Taken together, we would like to emphasize, that the use of fractality and *H* in particular, has two advantages which makes it a promising biomarker in the early detection of disease: (i) the reference score is a concrete number (e.g., 1) the difference can be interpreted as a measure of the deviation from this reference state, (ii) in principle the assessment of *H* can be integrated in the (f)MRI clinical routine protocol subject to the availability of sufficiently long fMRI-BOLD sequences. However, consistent with earlier observations with various fractal time series methods (Eke et al., 2000) -, as specifically stated by Riley et al. (2012), “AFA requires careful consideration of signal properties, parameter settings, and interpretation of results, and should not be applied blindly to unfamiliar signals.” (Riley et al., 2012). In line with earlier studies, our data showed the potential of fractal parameters in the detection of altered brain function in the clinical context. For that reason, it is highly recommended to follow up on the development of methods to making fractal analysis accessible to a wider public and delivering unambiguous results.

## Author contributions

SN, MR, and AS-B conceptualized and supervised the study, SN and AA acquired and analyzed the data. AS-B, MR, and KD supported the transfer of the findings into the current discussion of translational (AS-B) and clinical neuroscience (MR, KD). All authors contributed to and have approved the final manuscript.

### Conflict of interest statement

The authors declare that the research was conducted in the absence of any commercial or financial relationships that could be construed as a potential conflict of interest. The handling editor and reviewer AE declared their involvement as co-editors in the Research Topic, and confirm the absence of any other collaboration.

## Supplementary material

Figure S1 The scaling function f and the corresponding regression line of the entire signal (left upper row) and for its LFC only (right upper row). Please note that data points in the case of the LFC are linearly fitted, whereas this is not the case in the entire signal. The diagramm in the lower row shows that the slopes H of the regression lines are q-indepenent (monofractal). For this diagramm, MFDFA has been used (Ihlen, 2012). For demonstration, the example of a representative individual time course has been used.

Supplementary Presentation 1 Plots of raw BOLD series.

Supplementary Table 1 Bootstrap.

Supplementary Datasheet 1 Permutations.

## Abbreviations

BOLD: Blood-Oxygen-Level-Dependent
AFA: adaptive fractal analysis
dlPFC: dorsolateral prefrontal cortex
fMRI: functional magnetic resonance imaging
highImp: high impulsive
HFC: high frequency components
HC: hippocampus
*H*: Hurst Exponent
LFC: low frequency components
lowImp: low impulsive subjects
MFG: middle frontal gyrus
NAcc: nucleus accumbens
PSD: power spectrum density
rs-fMRI: resting-state fMRI
ROI: Regions of Interest
vmPFC: ventromedial prefrontal cortex.

## References

[B1] Bai F. Zhang Z. Yu H. Shi Y. Yuan Y. Zhu W.. (2008). Default-mode network activity distinguishes amnestic type mild cognitive impairment from healthy aging: a combined structural and resting-state functional MRI study. Neurosci. Lett. 438, 111–115. 10.1016/j.neulet.2008.04.021 18455308

[B2] Bak P. Tang C. Wiesenfeld K. (1987). Self-organized criticality: An explanation of the 1/f noise. Phys. Rev. Lett. 59, 381–384. 10.1103/PhysRevLett.59.381 10035754

[B3] Ball G. Stokes P. R. Rhodes R. A. Bose S. K. Rezek I. Wink A. M.. (2011). Executive functions and prefrontal cortex: a matter of persistence? Front. Syst. Neurosci. 5:3. 10.3389/fnsys.2011.00003 21286223 PMC3031025

[B4] Bari A. Robbins T. W. (2013). Inhibition and impulsivity: behavioral and neural basis of response control. Prog. Neurobiol. 108, 44–79. 10.1016/j.pneurobio.2013.06.005 23856628

[B5] Barnes A. Bullmore E. T. Suckling J. (2009). Endogenous human brain dynamics recover slowly following cognitive effort. PLoS ONE 4:e6626. 10.1371/journal.pone.0006626 19680553 PMC2721686

[B6] Benjamini Y. Hochberg Y. (1995). Controlling the false discovery rate: a practical and powerful approach to multiple testing. J. R. Stat. Soc. B 57, 289–300.

[B7] Boehler C. N. Appelbaum L. G. Krebs R. M. Hopf J. M. Woldorff M. G. (2010). Pinning down response inhibition in the brain–conjunction analyses of the Stop-signal task. Neuroimage 52, 1621–1632. 10.1016/j.neuroimage.2010.04.276 20452445 PMC2910135

[B8] Braver T. S. (2012). The variable nature of cognitive control: a dual mechanisms framework. Trends Cogn. Sci. 16, 106–113. 10.1016/j.tics.2011.12.010 22245618 PMC3289517

[B9] Brett M. Anton J.-L. Valabregue R. Poline J.-P. (2002). Region of interest analysis using an SPM toolbox [abstract], in Neuroimage International Conference on Functional Mapping of the Human Brain (Sendai).

[B10] Bullmore E. Barnes A. Bassett D. S. Fornito A. Kitzbichler M. Meunier D. (2009). Generic aspects of complexity in brain imaging data and other biological systems. Neuroimage 47:1125 10.1016/j.neuroimage.2009.05.032 19460447

[B11] Burnett Heyes S. Adam R. J. Urner M. Van Der Leer L. Bahrami B. Bays P. M.. (2012). Impulsivity and rapid decision-making for reward. Front. Psychol. 3:153. 10.3389/fpsyg.2012.00153 22661960 PMC3357492

[B12] Calhoun V. D. De Lacy N. (2017). Ten key observations on the analysis of resting-state functional MR imaging data using independent component analysis. Neuroimaging Clin. N. Am. 27, 561–579. 10.1016/j.nic.2017.06.012 28985929 PMC5657522

[B13] Cannon M. J. Percival D. B. Caccia D. C. Raymond G. M. Bassingthwaighte J. B. (1997). Evaluating scaled windowed variance methods for estimating the Hurst coefficient of time series. Phys. A 241:606 10.1016/S0378-4371(97)00252-5 PMC3204962 22049250

[B14] Carver C. S. Johnson S. L. Joormann J. (2008). Serotonergic function, two-mode models of self-regulation, and vulnerability to depression: what depression has in common with impulsive aggression. Psychol. Bull. 134, 912–943. 10.1037/a0013740 18954161 PMC2847478

[B15] Chambers C. D. Garavan H. Bellgrove M. A. (2009). Insights into the neural basis of response inhibition from cognitive and clinical neuroscience. Neurosci. Biobehav. Rev. 33, 631–646. 10.1016/j.neubiorev.2008.08.016 18835296

[B16] Chantiluke K. Halari R. Simic M. Pariante C. M. Papadopoulos A. Giampietro V.. (2012). Fronto-striato-cerebellar dysregulation in adolescents with depression during motivated attention. Biol. Psychiatry 71, 59–67. 10.1016/j.biopsych.2011.09.005 22015111

[B17] Churchill N. W. Spring R. Grady C. Cimprich B. Askren M. K. Reuter-Lorenz P. A.. (2016). The suppression of scale-free fMRI brain dynamics across three different sources of effort: aging, task novelty and task difficulty. Sci. Rep. 6:30895. 10.1038/srep30895 27498696 PMC4976369

[B18] Citrome L. (2015). A primer on binge eating disorder diagnosis and management. CNS Spectr. 20(Suppl. 1), 44–50; quiz 51. 10.1017/S1092852915000772 26683528

[B19] Ciuciu P. Abry P. He B. J. (2014). Interplay between functional connectivity and scale-free dynamics in intrinsic fMRI networks. Neuroimage 95, 248–263. 10.1016/j.neuroimage.2014.03.047 24675649 PMC4043862

[B20] Ciuciu P. Varoquaux G. Abry P. Sadaghiani S. Kleinschmidt A. (2012). Scale-free and multifractal time dynamics of fMRI signals during rest and task. Front. Physiol. 3:186. 10.3389/fphys.2012.00186 22715328 PMC3375626

[B21] Dalley J. W. Everitt B. J. Robbins T. W. (2011). Impulsivity, compulsivity, and top-down cognitive control. Neuron 69, 680–694. 10.1016/j.neuron.2011.01.020 21338879

[B22] Davis F. C. Knodt A. R. Sporns O. Lahey B. B. Zald D. H. Brigidi B. D.. (2013). Impulsivity and the modular organization of resting-state neural networks. Cereb. Cortex 23, 1444–1452. 10.1093/cercor/bhs126 22645253 PMC3643719

[B23] Deserno L. Wilbertz T. Reiter A. Horstmann A. Neumann J. Villringer A.. (2015). Lateral prefrontal model-based signatures are reduced in healthy individuals with high trait impulsivity. Transl. Psychiatry 5:e659. 10.1038/tp.2015.139 26460483 PMC4930122

[B24] Dona O. Noseworthy M. D. Dematteo C. Connolly J. F. (2017). Fractal Analysis of Brain Blood Oxygenation Level Dependent (BOLD) signals from children with mild traumatic brain injury (mTBI). PLoS ONE 12:e0169647. 10.1371/journal.pone.0169647 28072842 PMC5224975

[B25] Donnelly N. A. Holtzman T. Rich P. D. Nevado-Holgado A. J. Fernando A. B. Van Dijck G.. (2014). Oscillatory activity in the medial prefrontal cortex and nucleus accumbens correlates with impulsivity and reward outcome. PLoS ONE 9:e111300. 10.1371/journal.pone.0111300 25333512 PMC4205097

[B26] Eke A. Herman P. Bassingthwaighte J. B. Raymond G. M. Percival D. B. Cannon M.. (2000). Physiological time series: distinguishing fractal noises from motions. Pflugers Arch. 439, 403–415. 10.1007/s004249900135 10678736

[B27] Eke A. Herman P. Hajnal M. (2006). Fractal and noisy CBV dynamics in humans: influence of age and gender. J. Cereb. Blood Flow Metab. 26, 891–898. 10.1038/sj.jcbfm.9600243 16292253

[B28] Eke A. Herman P. Kocsis L. Kozak L. R. (2002). Fractal characterization of complexity in temporal physiological signals. Physiol. Meas. 23, R1–38. 10.1088/0967-3334/23/1/201 11876246

[B29] Eke A. Herman P. Sanganahalli B. G. Hyder F. Mukli P. Nagy Z. (2012). Pitfalls in Fractal Time Series Analysis: fMRI BOLD as an Exemplary Case. Front. Physiol. 3:417. 10.3389/fphys.2012.00417 23227008 PMC3513686

[B30] El-Gaby M. Shipton O. A. Paulsen O. (2015). Synaptic plasticity and memory: new insights from hippocampal left-right asymmetries. Neuroscientist 21, 490–502. 10.1177/1073858414550658 25239943

[B31] Feja M. Hayn L. Koch M. (2014). Nucleus accumbens core and shell inactivation differentially affects impulsive behaviours in rats. Prog. Neuropsychopharmacol. Biol. Psychiatry 54, 31–42. 10.1016/j.pnpbp.2014.04.012 24810333

[B32] Fox M. D. Raichle M. E. (2007). Spontaneous fluctuations in brain activity observed with functional magnetic resonance imaging. Nat. Rev. Neurosci. 8, 700–711. 10.1038/nrn2201 17704812

[B33] Fox M. D. Snyder A. Z. Vincent J. L. Raichle M. E. (2007). Intrinsic fluctuations within cortical systems account for intertrial variability in human behavior. Neuron 56, 171–184. 10.1016/j.neuron.2007.08.023 17920023

[B34] Fox M. D. Zhang D. Snyder A. Z. Raichle M. E. (2009). The global signal and observed anticorrelated resting state brain networks. J. Neurophysiol. 101, 3270–3283. 10.1152/jn.90777.2008 19339462 PMC2694109

[B35] Gentili C. Cristea I. A. Ricciardi E. Vanello N. Popita C. David D.. (2017). Not in one metric: Neuroticism modulates different resting state metrics within distinctive brain regions. Behav. Brain Res. 327, 34–43. 10.1016/j.bbr.2017.03.031 28342970

[B36] Gentili C. Vanello N. Cristea I. David D. Ricciardi E. Pietrini P. (2015). Proneness to social anxiety modulates neural complexity in the absence of exposure: a resting state fMRI study using Hurst exponent. Psychiatry Res. 232, 135–144. 10.1016/j.pscychresns.2015.03.005 25882042

[B37] Gilden D. L. Hancock H. (2007). Response variability in attention-deficit disorders. Psychol. Sci. 18, 796–802. 10.1111/j.1467-9280.2007.01982.x 17760776

[B38] Goldberger A. L. Amaral L. A. Hausdorff J. M. Ivanov P. Peng C. K. Stanley H. E. (2002). Fractal dynamics in physiology: alterations with disease and aging. Proc. Natl. Acad. Sci. U.S.A. 99(Suppl. 1), 2466–2472. 10.1073/pnas.012579499 11875196 PMC128562

[B39] Gorges M. Roselli F. Muller H. P. Ludolph A. C. Rasche V. Kassubek J. (2017). Functional connectivity mapping in the animal model: principles and applications of resting-state fMRI. Front. Neurol. 8:200. 10.3389/fneur.2017.00200 28539914 PMC5423907

[B40] Goya-Maldonado R. Walther S. Simon J. Stippich C. Weisbrod M. Kaiser S. (2010). Motor impulsivity and the ventrolateral prefrontal cortex. Psychiatry Res. 183, 89–91. 10.1016/j.pscychresns.2010.04.006 20542670

[B41] Hahn T. Dresler T. Ehlis A. C. Pyka M. Dieler A. C. Saathoff C.. (2012). Randomness of resting-state brain oscillations encodes Gray's personality trait. Neuroimage 59, 1842–1845. 10.1016/j.neuroimage.2011.08.042 21889990

[B42] Hausdorff J. M. (2007). Gait dynamics, fractals and falls: finding meaning in the stride-to-stride fluctuations of human walking. Hum. Mov. Sci. 26, 555–589. 10.1016/j.humov.2007.05.003 17618701 PMC2267927

[B43] He B. J. (2011). Scale-free properties of the functional magnetic resonance imaging signal during rest and task. J. Neurosci. 31, 13786–13795. 10.1523/JNEUROSCI.2111-11.2011 21957241 PMC3197021

[B44] He B. J. (2014). Scale-free brain activity: past, present, and future. Trends Cogn. Sci. 18, 480–487. 10.1016/j.tics.2014.04.003 24788139 PMC4149861

[B45] Herman P. Sanganahalli B. G. Hyder F. Eke A. (2011). Fractal analysis of spontaneous fluctuations of the BOLD signal in rat brain. Neuroimage 58, 1060–1069. 10.1016/j.neuroimage.2011.06.082 21777682 PMC3705180

[B46] Hinshaw S. P. (2017). Attention Deficit Hyperactivity Disorder (ADHD): controversy, developmental mechanisms, and multiple levels of analysis. Annu Rev Clin Psychol. 4:291–316. 10.1146/annurev-clinpsy-050817-084917 29220204

[B47] Hinshaw S. P. Carte E. T. Sami N. Treuting J. J. Zupan B. A. (2002). Preadolescent girls with attention-deficit/hyperactivity disorder: II. Neuropsychological performance in relation to subtypes and individual classification. J. Consult. Clin. Psychol. 70, 1099–1111. 12362960 10.1037//0022-006x.70.5.1099

[B48] Huang-Pollock C. L. Nigg J. T. Halperin J. M. (2006). Single dissociation findings of ADHD deficits in vigilance but not anterior or posterior attention systems. Neuropsychology 20, 420–429. 10.1037/0894-4105.20.4.420 16846260

[B49] Ihlen E. A. (2012). Introduction to multifractal detrended fluctuation analysis in matlab. Front. Physiol. 3:141. 10.3389/fphys.2012.00141 22675302 PMC3366552

[B50] Ihlen E. A. Vereijken B. (2010). Interaction-dominant dynamics in human cognition: beyond 1/fα fluctuation. J. Exp. Psychol. 139, 436–463. 10.1037/a0019098 20677894

[B51] Ivanov P. Ma Q. D. Bartsch R. P. Hausdorff J. M. Nunes Amaral L. A. Schulte-Frohlinde V. (2009). Levels of complexity in scale-invariant neural signals. Phys. Rev. E Stat. Nonlin. Soft Matter Phys. 79:041920 10.1103/PhysRevE.79.041920 19518269 PMC6653582

[B52] Ivanov P. C. Amaral L. A. N. Goldberger A. L. Havlin S. (1999). Multifractality in human heartbeat dynamics. Nature 399:461 10.1038/20924 10365957

[B53] Ivanov P. C. Nunes Amaral L. A. Goldberger A. L. Havlin S. Rosenblum M. G. Stanley H. E.. (2001). From 1/f noise to multifractal cascades in heartbeat dynamics. Chaos 11, 641–652. 10.1063/1.1395631 12779503

[B54] Kandel E. R. Schwartz J. H. Jessel T. M. (2000). Principles of Neuroscience, 4th Edn. New York, NY: McGraw Hill.

[B55] Keshner M. (1982). 1/f noise. Proc. IEEE 70, 212–218. 10.1109/PROC.1982.12282

[B56] Koch H. V. (1904). Sur une courbe continue sans tangente, obtenue par une construction géométrique élémentaire. Astron. och Fys. 1, 681–702.

[B57] Koch H. V. (1906). Une méthode géométrique élémentaire pour l'étude de certaines questions de la théorie des courbes planes. Acta Math. 30, 145–174. 10.1007/BF02418570

[B58] Lei X. Zhao Z. Chen H. (2013). Extraversion is encoded by scale-free dynamics of default mode network. Neuroimage 74, 52–57. 10.1016/j.neuroimage.2013.02.020 23454049

[B59] Li N. Ma N. Liu Y. He X. S. Sun D. L. Fu X. M.. (2013). Resting-state functional connectivity predicts impulsivity in economic decision-making. J. Neurosci. 33, 4886–4895. 10.1523/JNEUROSCI.1342-12.2013 23486959 PMC6618998

[B60] Liégeois R. Laumann T. O. Snyder A. Z. Zhou J. Yeo B. T. T. (2017). Interpreting temporal fluctuations in resting-state functional connectivity MRI. Neuroimage 10.1016/j.neuroimage.2017.09.012 28916180

[B61] Linkenkaer-Hansen K. (2002). Self Organized Criticality and Stochastic Resonance in the Human Brain Dept Of Engineering Physics and Mathematics. Helsinki: Helsinki University of Technology.

[B62] Lipsitz L. A. (2002). Dynamics of stabilitythe physiologic basis of functional health and frailty. J. Gerontol. 57, B115–B125. 10.1093/gerona/57.3.B115 11867648

[B63] Lipsitz L. A. Goldberger A. L. (1992). Loss of complexity and aging: potential applications of fractals and chaos theory to senescence. JAMA 267, 1806–1809. 10.1001/jama.1992.03480130122036 1482430

[B64] Lo C. C. Bartsch R. P. Ivanov P. C. (2013). Asymmetry and basic pathways in sleep-stage transitions. Europhys. Lett. 102:10008 10.1209/0295-5075/102/10008 24653582 PMC3956650

[B65] Mandelbrot B. (1967). How long is the coast of britain? statistical self-similarity and fractional dimension. Science 156, 636–638. 10.1126/science.156.3775.636 17837158

[B66] Mandelbrot B. B. (1983). The Fractal Geometry of Nature. New York, NY: W. H. Freeman.

[B67] Maxim V. Sendur L. Fadili J. Suckling J. Gould R. Howard R.. (2005). Fractional gaussian noise, functional MRI and Alzheimer's disease. Neuroimage 25, 141–158. 10.1016/j.neuroimage.2004.10.044 15734351

[B68] Mechelmans D. J. Strelchuk D. Donamayor-Alonso N. Banca P. Robbins T. W. Baek K.. (2017). Reward sensitivity and waiting impulsivity: shift towards reward valuation away from action control. Int. J. Neuropsychopharmacol. 20, 971–978. 10.1093/ijnp/pyx072 29020291 PMC5716204

[B69] Moreno-López L. Soriano-Mas C. Delgado-Rico E. Rio-Valle J. S. Verdejo-Garcia A. (2012). Brain structural correlates of reward sensitivity and impulsivity in adolescents with normal and excess weight. PLoS ONE 7:e49185. 10.1371/journal.pone.0049185 23185306 PMC3504042

[B70] Morris L. S. Kundu P. Baek K. Irvine M. A. Mechelmans D. J. Wood J.. (2016). Jumping the gun: mapping neural correlates of waiting impulsivity and relevance across alcohol misuse. Biol. Psychiatry 79, 499–507. 10.1016/j.biopsych.2015.06.009 26185010 PMC4764648

[B71] Nagy Z. Mukli P. Herman P. Eke A. (2017). Decomposing multifractal crossovers. Front. Physiol. 8:533. 10.3389/fphys.2017.00533 28798694 PMC5527813

[B72] Neufang S. Akhrif A. Herrmann C. G. Drepper C. Homola G. A. Nowak J.. (2016). Serotonergic modulation of 'waiting impulsivity' is mediated by the impulsivity phenotype in humans. Transl. Psychiatry 6:e940. 10.1038/tp.2016.210 27824354 PMC5314122

[B73] Penn A. A. Shatz C. J. (1999). Brain waves and brain wiring: the role of endogenous and sensory-driven neural activity in development. Pediatr. Res. 45, 447–458. 10.1203/00006450-199904010-00001 10203134

[B74] Qiu Y. W. Han L. J. Lv X. F. Jiang G. H. Tian J. Z. Zhuo F. Z.. (2011). Regional homogeneity changes in heroin-dependent individuals: resting-state functional MR imaging study. Radiology 261, 551–559. 10.1148/radiol.11102466 21875854

[B75] Riley M. A. Bonnette S. Kuznetsov N. Wallot S. Gao J. (2012). A tutorial introduction to adaptive fractal analysis. Front. Physiol. 3:371. 10.3389/fphys.2012.00371 23060804 PMC3460370

[B76] Robinson E. S. Eagle D. M. Economidou D. Theobald D. E. Mar A. C. Murphy E. R.. (2009). Behavioural characterisation of high impulsivity on the 5-choice serial reaction time task: specific deficits in 'waiting' versus 'stopping'. Behav. Brain Res. 196, 310–316. 10.1016/j.bbr.2008.09.021 18940201

[B77] Rösler M. Retz W. Retz-Junginger P. Stieglitz R. D. Kessler H. Reimherr F.. (2008). ADHS-Diagnose bei Erwachsenen. Nervenarzt 79, 320–327. 10.1007/s00115-007-2375-0 18210051

[B78] Sala M. Caverzasi E. Lazzaretti M. Morandotti N. De Vidovich G. Marraffini E.. (2011). Dorsolateral prefrontal cortex and hippocampus sustain impulsivity and aggressiveness in borderline personality disorder. J. Affect. Disord. 131, 417–421. 10.1016/j.jad.2010.11.036 21211852

[B79] Scheres A. Hamaker E. L. (2010). What we can and cannot conclude about the relationship between steep temporal reward discounting and hyperactivity-impulsivity symptoms in attention-deficit/hyperactivity disorder. Biol. Psychiatry 68, e17–e18. 10.1016/j.biopsych.2010.05.021 20615495

[B80] Sebastian A. Jacob G. Lieb K. Tuscher O. (2013). Impulsivity in borderline personality disorder: a matter of disturbed impulse control or a facet of emotional dysregulation? Curr. Psychiatry Rep. 15:339. 10.1007/s11920-012-0339-y 23424747

[B81] Sejdić E. Lipsitz L. A. (2013). Necessity of noise in physiology and medicine. Comput. Methods Programs Biomed. 111, 459–470. 10.1016/j.cmpb.2013.03.014 23639753 PMC3987774

[B82] Smitha K. A. Akhil Raja K. Arun K. M. Rajesh P. G. Thomas B. Kapilamoorthy T. R.. (2017). Resting state fMRI: A review on methods in resting state connectivity analysis and resting state networks. Neuroradiol. J. 30, 305–317. 10.1177/1971400917697342 28353416 PMC5524274

[B83] Sokunbi M. O. Gradin V. B. Waiter G. D. Cameron G. G. Ahearn T. S. Murray A. D.. (2014). Nonlinear complexity analysis of brain FMRI signals in schizophrenia. PLoS ONE 9:e95146. 10.1371/journal.pone.0095146 24824731 PMC4019508

[B84] Solanto M. V. Gilbert S. N. Raj A. Zhu J. Pope-Boyd S. Stepak B.. (2007). Neurocognitive functioning in AD/HD, predominantly inattentive and combined subtypes. J. Abnorm. Child Psychol. 35, 729–744. 10.1007/s10802-007-9123-6 17629724 PMC2265203

[B85] Stadnitski T. (2012). Measuring fractality, in Fractal Analyses: Statistical and Methodological Innovations and Best Practices, eds Holden J. G. Riley M. A. Gao J. Torre K. (Lausanne, CH: Frontiers in Physiology), 22–34. 10.3389/fphys.2013.00097 PMC3647382 23658545

[B86] Suckling J. Wink A. M. Bernard F. A. Barnes A. Bullmore E. (2008). Endogenous multifractal brain dynamics are modulated by age, cholinergic blockade and cognitive performance. J. Neurosci. Methods 174, 292–300. 10.1016/j.jneumeth.2008.06.037 18703089 PMC2590659

[B87] Thurner S. Windischberger C. Moser E. Walla P. Barth M. (2003). Scaling laws and persistence in human brain activity. Phys. A Statist. Mech. Appl. 326, 511–521. 10.1016/S0378-4371(03)00279-6

[B88] Voon V. Irvine M. A. Derbyshire K. Worbe Y. Lange I. Abbott S.. (2014). Measuring “waiting” impulsivity in substance addictions and binge eating disorder in a novel analogue of rodent serial reaction time task. Biol. Psychiatry 75, 148–155. 10.1016/j.biopsych.2013.05.013 23790224 PMC3988873

[B89] Weinstein A. M. (2017). An update overview on brain imaging studies of internet gaming disorder. Front. Psychiatry 8:185. 10.3389/fpsyt.2017.00185 29033857 PMC5626837

[B90] Wijnants M. L. Cox R. F. Hasselman F. Bosman A. M. Van Orden G. (2012). Does sample rate introduce an artifact in spectral analysis of continuous processes? Front. Physiol. 3:495. 10.3389/fphys.2012.00495 23346058 PMC3549522

[B91] Wink A. M. Bullmore E. Barnes A. Bernard F. Suckling J. (2008). Monofractal and multifractal dynamics of low frequency endogenous brain oscillations in functional MRI. Hum. Brain Mapp. 29, 791–801. 10.1002/hbm.20593 18465788 PMC6870616

[B92] Zhang D. Snyder A. Z. Fox M. D. Sansbury M. W. Shimony J. S. Raichle M. E. (2008). Intrinsic functional relations between human cerebral cortex and thalamus. J. Neurophysiol. 100, 1740–1748. 10.1152/jn.90463.2008 18701759 PMC2576214

