# Supplementary figures and images for: Fractal Analysis of BOLD Time Series in a Network Associated With Waiting Impulsivity
Source: Front Physiol. 2018 Oct 4;9:1378. doi: 10.3389/fphys.2018.01378 (PMC6180197; doi:10.3389/fphys.2018.01378)

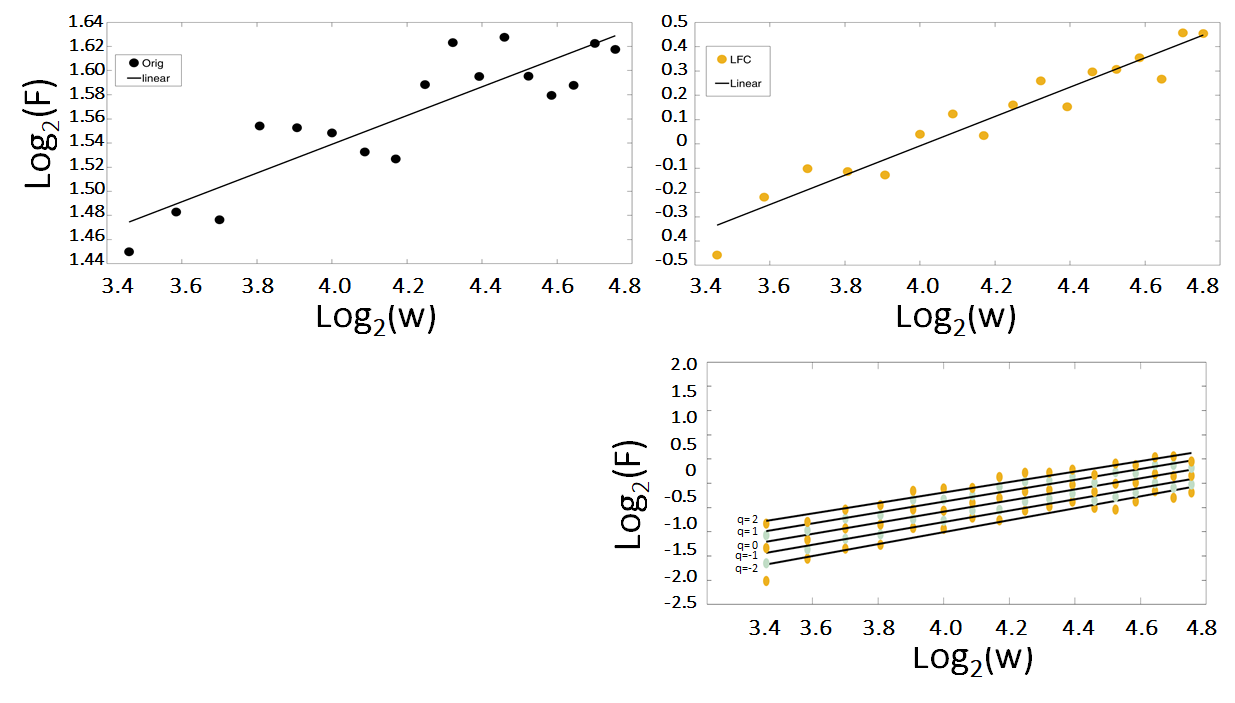

Supplement: Figure S1 — The scaling function f and the corresponding regression line of the entire signal (left upper row) and for its LFC only (right upper row). Please note that data points in the case of the LFC are linearly fitted, whereas this is not the case in the entire signal. The diagramm in the lower row shows that the slopes H of the regression lines are q-indepenent (monofractal). For this diagramm, MFDFA has been used (Ihlen, 2012). For demonstration, the example of a representative individual time course has been used. [file Image_1.TIF]

## Slide 1
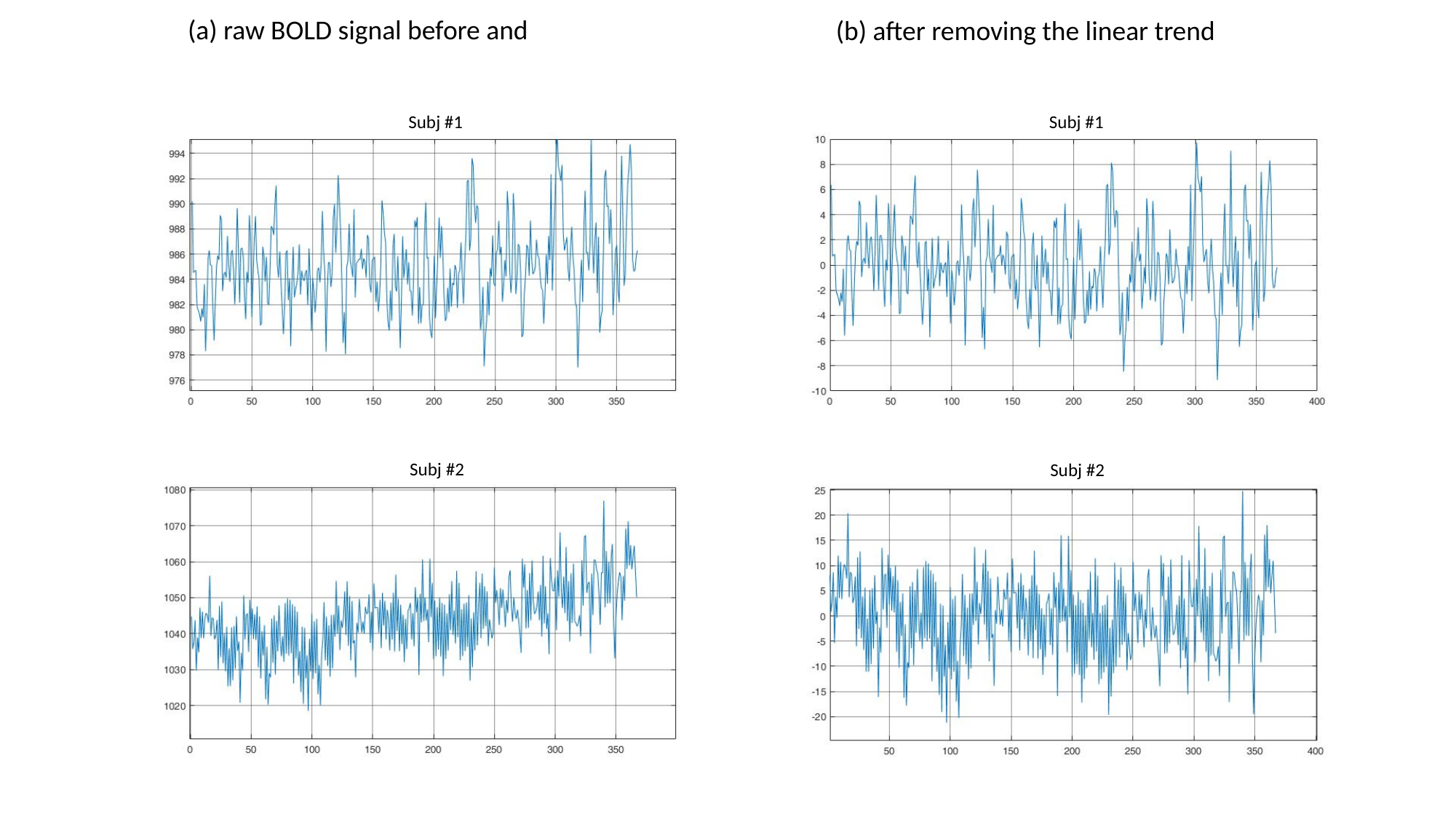

(a) raw BOLD signal before and
(b) after removing the linear trend
Subj #1
Subj #1
Subj #2
Subj #2

Supplement: Supplementary Presentation 1 — Plots of raw BOLD series. [file Presentation_1.PPTX]
