# Supplementary material for: Fractal Analysis of BOLD Time Series in a Network Associated With Waiting Impulsivity
Source: Front Physiol. 2018 Oct 4;9:1378. doi: 10.3389/fphys.2018.01378 (PMC6180197; doi:10.3389/fphys.2018.01378)
Supplement: Supplementary Table 1 — Bootstrap. [file Table_1.DOCX]

|  | **lowImp [n=66]** | | **highImp [n=38]** | |  |
| --- | --- | --- | --- | --- | --- |
| **T A S K** | | | | |  |
|  | **lower bound** | **upper bound** | **lower bound** | **upper bound** | |
| **rHC** | .85 | .91 | .84 | .91 | |
| **lHC** | .89 | .94 | .85 | .91 | |
| **lMFG** | .92 | .97 | .86 | .93 | |
| **rMFG** | .90 | .97 | .86 | .93 | |
| **ACC** | **.93** | **.99** | **.86** | **.91** | |
| **rNAcc** | **.90** | **.96** | **.83** | **.89** | |
| **lAMY** | .86 | .92 | .83 | .89 | |
| **vmPFC** | .96 | 1.0 | .94 | 1.0 | |
| **R E S T** | | | | |  |
|  | **lower** | **upper** | **lower** | **upper** | |
| **rHC** | .91 | .97 | .88 | .95 | |
| **lHC** | .93 | .99 | .91 | .98 | |
| **lMFG** | .99 | 1.0 | .97 | 1.0 | |
| **rMFG** | .97 | 1.0 | .96 | 1.0 | |
| **ACC** | .99 | 1.0 | .97 | 1.0 | |
| **rNAcc** | .95 | 1.0 | .91 | .99 | |
| **lAMY** | .89 | .95 | .88 | .95 | |
| **vmPFC** | 1.0 | 1.1 | 1.0 | 1.1 | |

bootstrapping was performed using SPSS, number of samples was 1000, confidence interval level (%) were 95, and they were bias corrected accelerated (BCa)
